# Breast cancer morbidity and mortality in rural Ethiopia: data from 788 verbal autopsies

**DOI:** 10.1186/s12905-022-01672-7

**Published:** 2022-03-24

**Authors:** Wondimu Ayele, Amand Führer, Gabriele Anna Braun, Franziska Formazin, Andreas Wienke, Lesley Taylor, Susanne Unverzagt, Adamu Addissie, Eva J. Kantelhardt

**Affiliations:** 1 School of Public Health Department of Biostatistics and Epidemiology, Addis Ababa University, Addis Ababa, Ethiopia; 2 Institute for Medical Epidemiology, Biometrics and Informatics, Interdisciplinary Center for Health Sciences, Martin-Luther-University Halle-Wittenberg, Magdeburgerstr. 8, 06097 Halle (Saale), Germany; 3 City of Hope National Medical Center, Duarte, CA USA; 4 Center of Health Sciences, Institute of General Practice and Family Medicine, Martin-Luther-Universitat Halle-Wittenberg, Halle, Germany

**Keywords:** Breast cancer, Cause of death, Verbal autopsy, Duration of illness

## Abstract

**Introduction:**

In Ethiopia, breast cancer is the leading cause of cancer among women. Little is known about the duration of disease and symptoms of patients who died from breast cancer in rural Ethiopia. The objective of this study was to assess breast cancer mortality with a particular focus on the self-reported duration of illness including suffering of symptoms, and need for medical care.

**Methods:**

The cause of death was determined among randomly selected Ethiopian women residing in western Ethiopia. A modified standard verbal autopsy (VA) questionnaire was completed by women whose sisters had died. The questionnaires were reviewed by two independent local physicians to assign a cause of death. We analyzed pattern of cause of deaths, duration of suffering, symptoms, and treatment received.

**Result:**

In our study, the age at death was very similar to other population-based data from Ethiopia. We found 32% of 788 deaths were caused by communicable diseases, 12.1% by neoplasms, and 9.4% by pregnancy/maternal mortality. Breast cancer was the second leading neoplasm, responsible for 21 (2.7%) of all deaths (95% CI 1.5–3.7%), and was among the top five causes of non-communicable deaths. The median age of breast cancer death was 37 years, younger than for other causes of death. The median duration of illness with breast cancer was around 1 year. This was substantially more compared to the duration of infectious diseases, but less than the duration of reproductive neoplasms, diabetes, and epilepsy.

**Discussion/conclusion:**

Breast cancer deaths are common causes of death in women of rural Ethiopia. When assessing the total duration of illness according to specific causes of death, breast and other cancers accounted for a large share of the burden. This has practical implications and highlights the need for palliative care for cancer patients. Substantial efforts are necessary to improve early detection and treatment for breast cancer to reduce premature death in women.

**Supplementary Information:**

The online version contains supplementary material available at 10.1186/s12905-022-01672-7.

## Introduction

Breast cancer deaths have become recognized as an emerging public health problem in developing countries in the past few decades. Breast cancer is the most frequently diagnosed malignancy among women and the leading cause of cancer death among females worldwide, with an estimated 2.1 million cases and 627,000 deaths in 2018. Worldwide, breast cancer accounted for 11.5% of new cancer cases and 6.6% of deaths due to all cancers in 2018. The age-standardized breast cancer incidence and mortality rates in East Africa are 33.0 and 17.9 per 100,000 women per year, respectively [1].

There are striking differences in mortality rates from breast cancer in developed and developing countries attributed to late presentation and lack of therapy [2–5]. In most high-income countries, more than 70% of breast cancer patients are diagnosed in stages I and II. However, only 20–50% of patients in the majority of low- and middle-income countries are diagnosed at early stages [6–8]. The majority of breast cancer in women in low-income countries present at an advanced clinical stage, resulting in limited and difficult therapeutic options and contributing to the poor survival rate [6].

In Ethiopia, breast cancer is the leading cause of cancer-morbidity among adult women, accounting for one-third of all cancer cases among women and one in five of all cancer cases [8, 9]. An estimated 16,133 new breast cancer cases and 9061 breast cancer deaths occur annually in the country [1]. Women living in rural areas often seek treatment from traditional healers before seeking help within the formal health system. Only 4.5% of breast cancer patients initially seek care in a cancer hospital, whereas 70% of patients seek care first from a traditional healer or at a primary care site. In contrast, patients who have direct access to local and regional hospitals have the fewest number of encounters for treatment elsewhere [10].

The magnitude of mortality and duration of suffering from illness are important data to understand the burden of disease in a population. But in many low-income countries, no registration on the cause of death occurs due to a lack of medical death certificates or death registry. Verbal autopsy (VA) is a method using a questionnaire to assess details about signs and symptoms preceding the death and a physician's review to conclude a likely cause of death [11]. This is done at sentinel sites to obtain information on mortality within the community. This study used verbal autopsy data to obtain information on the magnitude of breast cancer mortality and the duration of illness, compared to other causes of death in a rural part of Western Ethiopia. This data aims to reflect morbidity and mortality due to breast cancer in rural Ethiopia with a focus on the self-reported duration of the illness considering time with breast symptoms, pain, and under medical care and show the need for specific health services of the disease commonly not considered relevant in the setting.

## Methods

### Study area and period

For this analysis, data were obtained from three prospective verbal autopsy surveys, conducted between 2011 and 2012 in the West Wellega (Aira, Guliso, Begi, Kondala), Gidami (Kelem) and Bale districts of the Oromia region, Ethiopia, with an estimated 237,222 and 1,402,492 inhabitants respectively [12].

### Study design

A community-based cross-sectional study was carried out and modified standard verbal autopsy questionnaires were completed in interviews with randomly selected female relatives who confirmed they had a deceased sister. Structured data collection tools were used to obtain information about the deceased sisters of the respondents [13].

### Study population

The study population comprised of all deceased sisters who had died in the 10 years preceding the survey to minimize recall bias. Information was obtained through interviews with their female relatives.

### Inclusion and exclusion criteria

All adult female relatives from participants who were at least 15 years old and died in the 10 years preceding the survey in Aira, Guliso, Begi, and Bale were included. Excluded from the analysis were any reported death of women under the age of 15 or any case in which interviewees were unable to describe the symptoms leading to the death of their family member.

### Sampling and sampling procedure

All three studies employed a similar approach for gathering data. All female residents in randomly selected clusters were interviewed about their relative's vital status utilizing the Direct Sisterhood Method [14]. If sisters died, caregivers/sisters were how long their female family member suffered from the disease prior to death and which symptoms she experienced.

### Sample size

We assumed three in five cancer deaths to be due to breast cancer. Therefore, to detect 3% with a precision of 1.75–4.25%, 800 verbal autopsies were needed [15].

### Data collection tools

A standard four-digit VA questionnaire on signs and symptoms, duration of illness, and health service visits was used for data collection [16]. Few modifications to the survey were made with input from an expert medical panel to include detailed breast cancer symptoms. These additional questions were intended to improve the questionnaire’s sensitivity towards malignant diseases and to facilitate a differential diagnosis among them.

### Operational definitions

#### The specific cause of death

The cause of death for the completed VA questionnaire was reviewed by two independent physicians who assigned an underlying cause of death. Intra-reviewer reliability was tested for 10% of the sample. A third physician review was added in case of contradicting cause of death.

#### Unknown cause of death

A death was classified as “unknown cause” when three independent local physicians assigned different causes of death or when the two first physicians classified the cause of death concordantly as “unknown”.

#### A broad category of cause of death

The cause of death was further broadly categorized or grouped using the WHO International Classification of Disease (ICD-10) code [17].

#### Duration of illness

The period between the date of death and the first manifestation of symptoms, pain, or other suffering from the disease or injury leading to death.

### Data quality management and analysis

The collected data were checked for completeness and accuracy and corrected before leaving the household. All data were entered into an electronic database, coded, and checked for missing values, outliers, and inconsistencies. The completed VA questionnaires were reviewed by two independent local physicians to assign three causes of death (underlying, immediate, and contributing factors) according to the WHO International Classification of Disease (ICD-10) code [17]. Differences in diagnoses between these physicians were subsequently reviewed by a third independent physician. The final cause of death bases on the agreement between any of the two physicians.

We performed descriptive statistics to calculate relative and absolute frequencies of causes of death and socio-demographic variables. We report relative frequencies and their 95% confidence intervals (CI) and medians with their interquartile range (IQR).

## Results

**Fig. 1:**
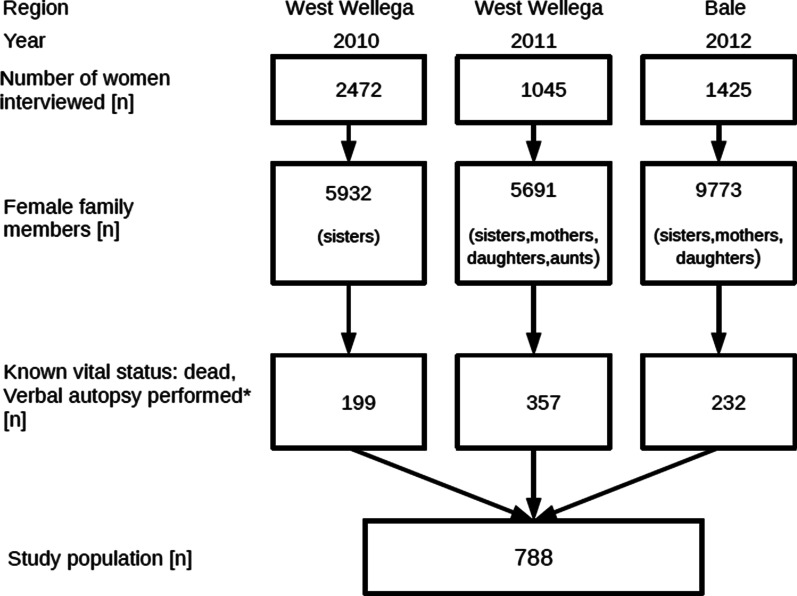
Consort diagram for study population

A total of 4942 women were interviewed and reported on about 21,396 female family members. Of them, 788 (3.7%) died at the age above 15 years between 2001 and 2012 (shown in Fig. 1). The physicians agreed in 67% of the VA code diagnoses, with intra-reviewer reliability of about 72% and 80%.

### Socio-demographic characteristics of patients who died from breast cancer

The sample’s age distribution (Additional file 1: Table S1) is similar to the age structure of adult women in the Health and demographic surveillance sites (HDSS) in Ethiopia [14]. Thus, we can assume the age distribution of deceased among our study sample is representative compared to other similar studies in Ethiopia (Additional file 1: Table S1). The median age of breast cancer deaths was 37 (IQR 26–45) years, which is substantially lower compared to the median age for deaths from other diseases (with a median of 46 (IQR 32–60) years). A total of 11 (52.3%) breast cancer deaths occurred among women between 15 and 49 years and eight (38.1%) women died in the agegroup between 50 and 64 years. All other socio-demographic information of women who died from breast cancer was comparable to non-breast cancer deaths (Table 1).

**Table 1:** Socio-demographic characteristics of adult females deceased between 2001 and 2012 in West Welega and Bale regions, Ethiopia, 2013

Socio-demographic variables	Breast cancer cause of death	Non-breast cancer cause of death
	n (%)	n (%)
Age group		
15–24	2 (9.5)	72 (9.4)
25–34	4 (19.0)	128 (16.7)
35–49	5 (23.8)	201 (26.2)
50–64	8 (38.1)	202 (26.3)
65+	2 (9.5)	164 (21.4)
Marital status		
Single	0 (0.0)	49 (6.4)
Married	17 (81.0)	550 (72.1)
Divorced	0 (0.0)	10 (1.3)
Widowed	4 (19.9)	141 (18.5)
Unknown	0 (0.0)	13 (1.7)
Educational status		
Illiterate	16 (76.2)	610 (81.9)
1–8 grade	3 (14.3)	90 (12.1)
9–12	2 (9.5)	43 (5.8)
12+	0 (0.0)	2 (0.3)
Occupation of the deceased		
Housewife or farmer	20 (95.2)	702 (92.2)
Trader	0 (0.0)	11 (1.4)
Employee	0 (0.0)	11 (1.4)
Other	0 (0.0)	22 (3.0)
Unknown	1 (4.8)	15 (2.0)
Place of death		
Home	19 (90.5)	624 (82.2)
Hospital	0 (0.0)	62 (8.2)
Other health facility	0 (0.0)	19 (2.5)
On the way to hospital	0 (0.0)	16 (2.1)
Unknown	2 (9.5)	27 (3.6)
Other	0 (0.0)	11 (1.4)

### Causes of death

**Fig. 2:**
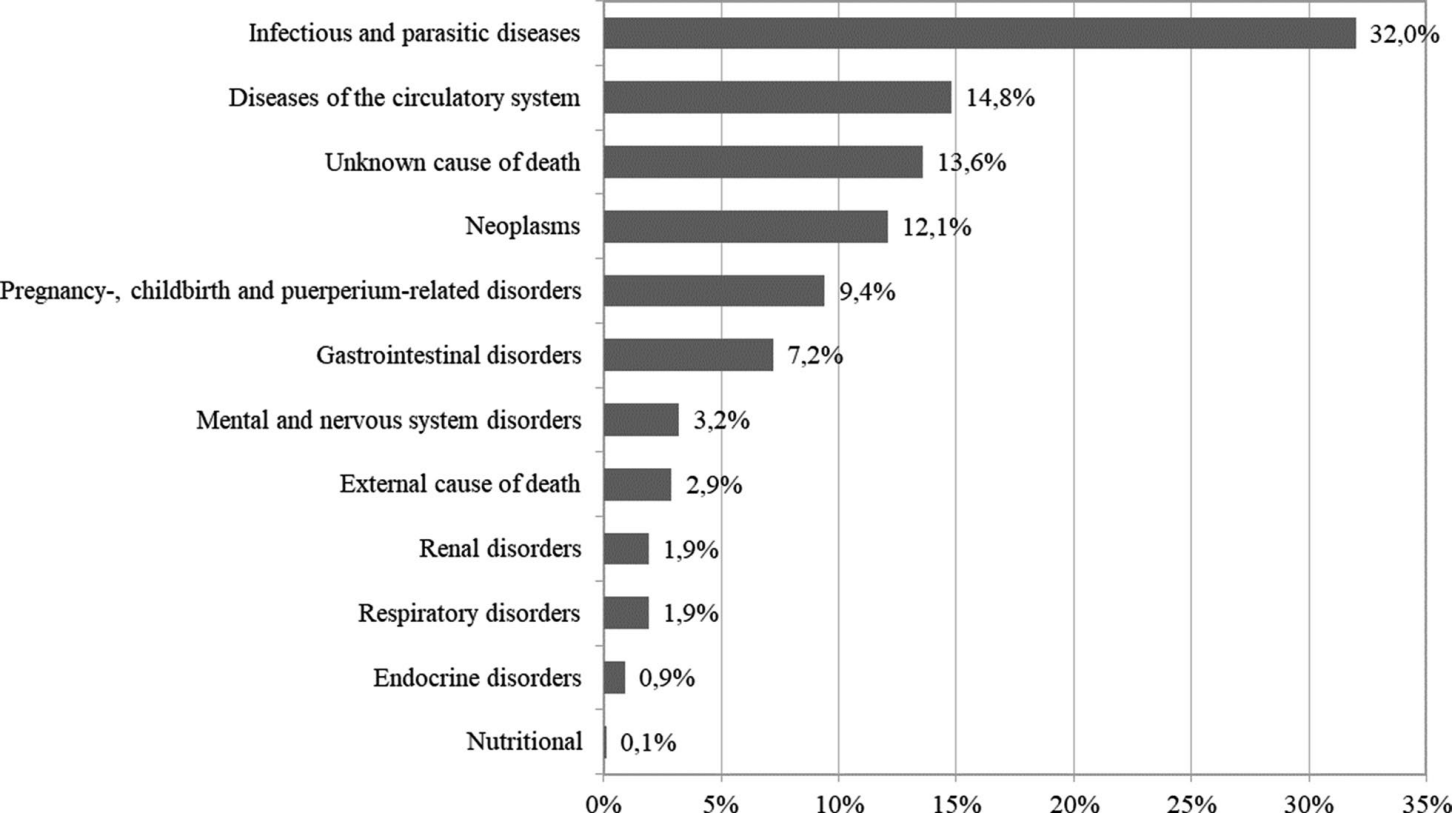
Adult female broad categories of death in three sites of Oromia, Ethiopia 2010–2012

In total, 32.0% (95% CI 28.7–35.2%) of all deaths occurred due to a communicable disease (Fig. 2). Most frequent specific causes of death were pulmonary tuberculosis (8.8%; 95% CI 6.8–10.7%), diarrhea (5.8%; 95% CI 4.2–7.5%), unspecified infectious diseases (5.7%; 95% CI 4.1–7.3%), malaria (5.2%; 95% CI 3.6–6.7%) and HIV/AIDS (4.3%; 95% CI 2.9–5.7%).

Non-communicable diseases (NCDs) accounted for around 42.1% of deaths (95% CI 38.7–45.6%). Most frequent NCDs were unspecified cardiac (7.5%; 95% CI 5.6–9.3%), stroke (6.1%; 95% CI 04.4–7.8%), digestive neoplasms (3.8%; 95% CI 2.5–5.1%), other unspecified gastrointestinal disorder (3.4%; 95% CI 2.1–4.7%), and breast cancer (2.7%; 95% CI 1.5–3.7%). Another 9.4% (95% CI 7.3–11.4%) of deaths were related to pregnancy and childbirth and 2.9% (95% CI 1.7–4.1%) due to external causes of death (shown in Fig. 2). Differences between the most frequent causes of death between the three studies are summarized in Additional file 2: Table S2.

### Breast cancer mortality, other causes of death, and duration of illness

The majority of infectious diseases (such as diarrhea, meningitis and encephalitis, malaria acute respiratory infection) and maternal causes of death had a median duration of illness of fewer than 7 days (Table 2).

**Table 2:** Duration of illness by specific cause of death at three sites of the Oromia region, Ethiopia 2010–2012

Cause of death	# (%)	Rank	Total number of days	Rank of total	Median (days)	IQR (days)
Cause of death unknown	107 (13.6)	1	30,363	1	62	14–365
Other and unspecified cardiac diseases	59 (7.5)	3	26,631	2	180	14–365
Pulmonary tuberculosis	69 (8.8)	2	23,065	3	255	62–365
Epilepsy	17 (2.2)	14	23,049	4	730	60–2190
Stroke	48 (6.1)	4	15,761	5	14	3–180
Reproductive neoplasms	12 (1.5)	19	11,802	6	730	365–1095
HIV/AIDS	34 (4.3)	8	10,489	7	365	61–365
Other unspecified Gastrointestinal disorders	27 (3.4)	11	10,399	8	150	60–700
Breast neoplasms	21 (2.7)	12	9296	9	365	210–730
Mental and nervous system disorders	8 (1.0)	24	8830	10	365	9–2555
Other and unspecified neoplasms	21 (2.7)	13	8661	11	195	16–548
Diabetes mellitus	7 (0.9)	26	7085	12	730	90–1825
Digestive neoplasms	30 (3.8)	10	6935	13	184	121–365
Other and unspecified infection diseases	45 (5.7)	6	6240	14	31	14–167
Renal failure	15 (1.9)	16	5471	15	90	7–365
Liver cirrhosis	14 (1.8)	17	5031	16	365	92–365
Diarrheal	46 (5.8)	5	4120	17	7	7–21
Chronic obstructive pulmonary disease	7 (0.9)	27	2250	18	548	60–1095
Respiratory neoplasms	10 (1.3)	22	1513	19	137	21–240
Other and unspecified maternal CoD	13 (1.6)	18	679	20	2	1–93
Asthma	8 (1.0)	25	459	21	45	3–365
Acute abdomen	16 (2.0)	15	416	22	2	1–7
Malaria	41 (5.2)	7	359	23	6	3–12
Obstetric hemorrhage	33 (4.2)	9	171	24	2	1–3
Acute cardiac disease	10 (1.3)	23	157	25	10	3–20
Acute respiratory infection	12 (1.5)	20	113	26	6	2–14
Pregnancy-related sepsis	7 (0.9)	28	100	27	5	5–15
Pregnancy-induced hypertension	6 (0.8)	29	69	28	12	6–14
Meningitis and encephalitis	5 (0.6)	30	53	29	7	5–7
Obstructed labor	12 (1.5)	21	34	30	2	2–4
Abortion-related death	2 (0.3)	34	17	31	9	3–14
Intentional self-harm	5 (0.6)	32	1	32	0	0–1
Road traffic accident	3 (0.4)	33	1	32	0	0–1
Other and unspecified external CoD	2 (0.3)	35	1	32	1	0–1
Other transport accident	2 (0.3)	36	1	32	0	0–1
Anemia of pregnancy	1 (0.1)	37	1	32	1	1–1
Accidental fall	5 (0.6)	31	0	33	0	0–1

In contrast, patients with NCDs suffered for a prolonged time from their illnesses, the longest duration being in the case of reproductive neoplasms (median 730 days), diabetes (730 days), epilepsy (730 days), chronic obstructive pulmonary disease (548 days), HIV/AIDS (365 days) and breast cancer (365 days). Seven in ten women suffered for more than 6 months from breast cancer.

### Major reported symptoms and treatment of patients with death due to breast cancer

**Fig. 3:**
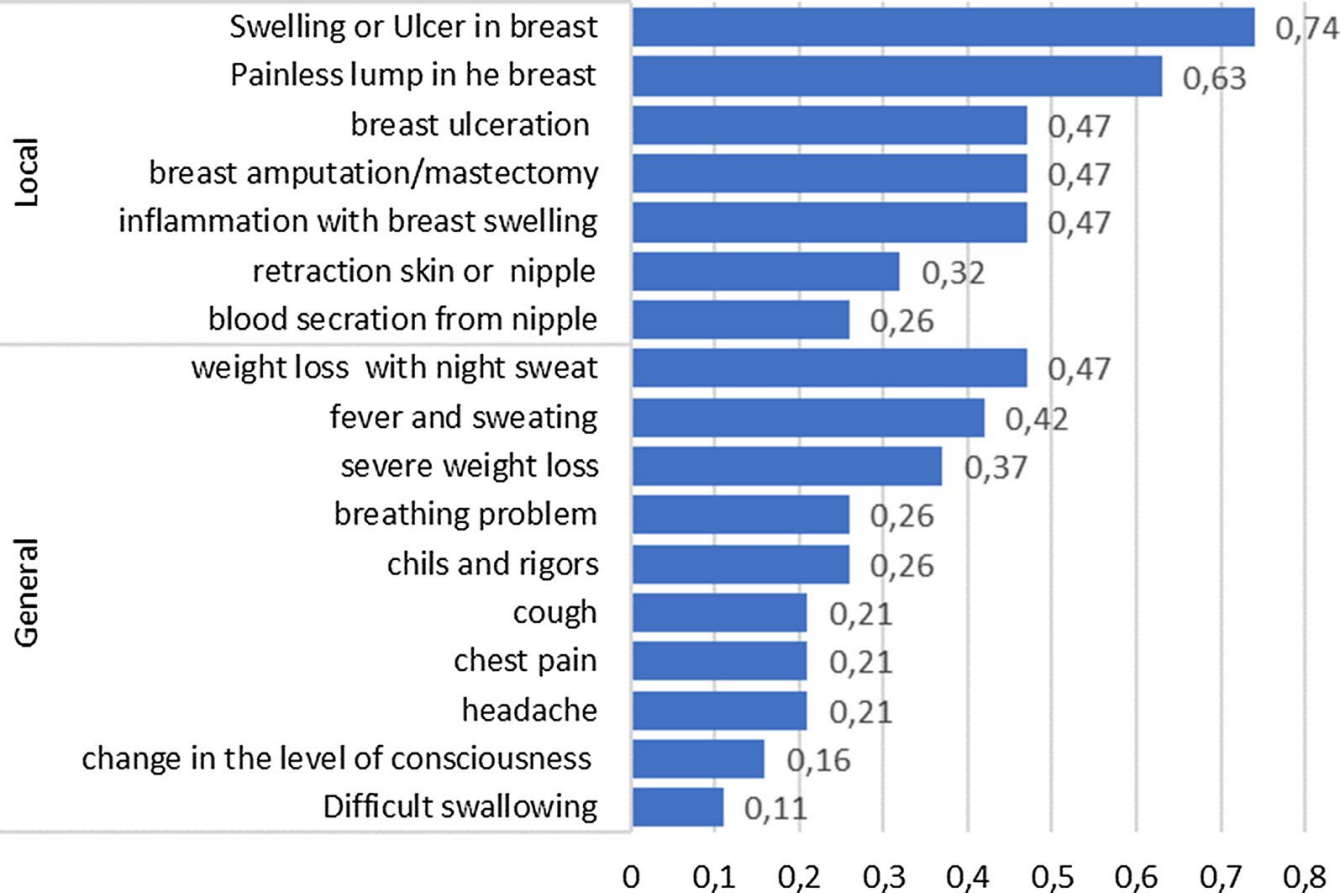
Major report symptoms of adult women deceased due to breast cancer cause of death

Around 74% of participants reported that the patient who died from breast cancer suffered from swelling or ulcers in the breast, 63% had a painless lump in the breast, and 47% had breast ulceration and/or inflammation with breast swelling (Fig. 3). About one-fourth of women with breast cancer had bloody nipple discharge. Regarding general symptoms, 47% of women had weight loss and night sweats, 42% had fever and sweating, and 26% had breathing problems.

Concerning seeking treatment, more than 68% of women with breast cancer were hospitalized at some point and received treatment (details were not specified) before death. About two-thirds of the patients had surgery and 47% had breast surgery or mastectomy. About 37% received antibiotics at some point during their care. Around 90.5% of women who died of breast cancer and 82.2% of women who died of other causes died at home.

## Discussion

The main aim of this study was to determine the pattern of breast cancer mortality and other causes of death among adult women in rural Ethiopia using verbal autopsy. We also assessed the duration of illness for each underlying cause of death with special attention to breast cancer morbidity.

In this study, the median age of adult women in rural Ethiopia who died from breast cancer was 37 years, which is considerably younger than the median age at death in this cohort. This age is considered young given the average lifespan of Ethiopian women (at the time of the survey about 61.1 years in 2010 [12]). Other studies revealed high proportions of breast cancer diagnosed in young age groups in Ethiopia, often with a late presentation. Often patients visit traditional healers, lack awareness of existing treatments, and experience delay at health facilities. All these factors negatively influence the prognosis of breast cancer in rural Ethiopia [14, 18]. This study found that the majority of breast cancer deaths occur among women in the reproductive age group. This implies that, in addition to attention on pregnancy- or childbirth-related deaths, awareness needs to be raised regarding breast cancer affecting women in this age group where they are responsible for families and children [16]. We found that breast cancer is the second leading cause of death from neoplasms in women and among the top five causes of death from NCD. Studies from other settings report that breast cancer is the leading cause of cancer death among adult women in developing countries [19, 20].

This study found that, in the rural setting of Ethiopia, NCD accounted for 42% of deaths among women, whereas infectious and parasitic causes of death accounted for 32% of deaths. Several studies align with our findings and have also reported that women in sub-Saharan countries, including Ethiopia, suffer from a double burden of non-communicable and infectious diseases [21–23]. This has implications to increase resource allocation.

The majority of women in this region of Ethiopia died at home (90.5%). This proportion was higher than in a study conducted in Addis Ababa, the capital of Ethiopia, which reported that 71.3% of breast cancer patients died at home. This could be explained by the fact that our study was conducted in a rural setting in Ethiopia where there is an underdeveloped infrastructure, limited health facilities, and low utilization and coverage of health services [24]. Moreover, it has been reported from other African countries such as Nigeria that long waiting times to receive treatment, limited health care resources, and e.g. socio-cultural norms, attitudes, and beliefs explain the observed high rate of women dying at home [25]. We found that most of the participants had undergone breast surgery only. Systemic treatment was not available in rural areas and travelling to Addis Ababa was reported impossible due to financial constraints.

Most women who died from breast cancer had symptoms of their illness for over 1 year prior to their death. In contrast, the median duration of suffering was 7 days or less for communicable diseases like malaria, acute respiratory infection, diarrhea, meningitis, and encephalitis, which accounted for 18% of all deaths in the population. The long duration of illness may affect household and community psycho-social and economic status in a number of ways. These may include disrupted family activities and productivity, anxiety or depression, interruption of social networks of support, high out-of-pocket costs for healthcare, and reductions in family savings and investments [26, 27]. These intertwined compounding economic effects can also be felt throughout communities, as within many African cultures, and particularly in Ethiopia, individuals with chronic illness usually receive support from family as well from the surrounding community [28]. Ethiopia made significant changes in maternal-child health and communicable diseases over the last two decades. However, less attention is still given to the prevention and control of noncommunicable diseases. The new Ethiopian cancer control and prevention strategy was drafted in 2016 and includes six new peripheral cancer centers. These centers are still not providing service. Therefore we believe the situation in rural Ethiopia has not changed much since 2012. Study on breast awareness and breast cancer in rural Ethiopia showed a high unmet need for breast care in a rural setting [29].

## Strengths and limitations

This is to our knowledge the first study to conduct surveillance in West Ethiopia in a population without previous observation. The physician's review result showed consistency in more than two-thirds of the VA code diagnoses with intra-reviewer reliabilities of about 72% and 80%, which was very similar to previous studies [11]. Additionally, this is the first study to compare the duration of illness in a considerable number of 788 deaths. Although the data is considerably old, it still presents the burden of breast cancer in a rural setting without access to a cancer center. The study had also some limitations, as sisters, mothers, and aunts were asked about a wide range of signs and symptoms that led to death in the last 10 years, which might have introduced recall bias. To avoid making relatives anxious, some patients may have not disclosed their symptoms to close family members.

We detected 13.6% of cases with an unknown cause of death, other studies reported around 10% unknown cause of death. Reliability and repeatability towards interpreting the VAs and assigning causes of death by physicians could be mentioned as a limitation but various other studies have used this methodology [30].

## Conclusion and recommendations

Non-communicable diseases including breast cancer are among the leading causes of women’s death in rural Ethiopia, breast cancer frequently occurs in a young reproductive age group, and results in a long duration of illness. These findings are an indication for more efforts to prevent non-communicable causes of death among women, in addition to infectious and maternal causes. The Ministry of Health should scale up preventive and curative interventions in rural parts of the country. The long duration of illness shows the urgent need for palliative care for cancer patients in rural areas. Substantial efforts are necessary to improve early detection and access to care for breast cancer patients to reduce suffering and premature deaths. Governmental and non-governmental organizations and health care providers should give serious attention to developing and implementing adequate breast cancer services within the primary health care system.

## Supplementary Information

**Additional file 1.** Age structure used on the study’s population compared to those of documented deaths at Kersa Health and Demographic surveillance site of Ethiopia.

**Additional file 2.** Top leading specific cause of death in three site of Oromia, Ethiopia 2010-2012.

## Data Availability

The datasets used and/or analysed during the current study available from the corresponding author on reasonable request.
