# Supplementary material for: Breast cancer morbidity and mortality in rural Ethiopia: data from 788 verbal autopsies
Source: BMC Womens Health. 2022 Mar 24;22:89. doi: 10.1186/s12905-022-01672-7 (PMC8951700; doi:10.1186/s12905-022-01672-7)
Supplement: Supplementary file 1 — Additional file 1. Age structure used on the study’s population compared to those of documented deaths at Kersa Health and Demographic surveillance site of Ethiopia. [file 12905_2022_1672_MOESM1_ESM.docx]

Supplement 1: Age structure used on the study´s population compared to those of documented deaths at Kersa Health and Demographic surveillance site of Ethiopia.

| Age | Study population | HDSS of Ethiopia |
| --- | --- | --- |
| 15-24 | 9.4% | 9.4% |
| 25-34 | 16.8% | 12.6% |
| 35-44 | 19.0% | 12.7% |
| 45-54 | 16.5% | 13.7% |
| 55-64 | 17.3% | 16.9% |
| 65+ | 21.1% | 35.4% |
