# Supplementary material for: Breast cancer morbidity and mortality in rural Ethiopia: data from 788 verbal autopsies
Source: BMC Womens Health. 2022 Mar 24;22:89. doi: 10.1186/s12905-022-01672-7 (PMC8951700; doi:10.1186/s12905-022-01672-7)
Supplement: Supplementary file 2 — Additional file 2. Top leading specific cause of death in three site of Oromia, Ethiopia 2010-2012. [file 12905_2022_1672_MOESM2_ESM.docx]

Supplement 2: Top leading specific cause of death in three site of Oromia, Ethiopia 2010-2012.

|  | Avira region | Guliso region | Begi region | Total |
| --- | --- | --- | --- | --- |
|  | #(%) | #(%) | #(%) | #(%) |
| Pulmonary Tuberculosis | 14(7.0) | 27(7.6) | 28(12.1) | 69(8.8) |
| Unspecified cardiac | 20(10.1) | 29(8.1) | 10(4.3) | 59(7.5) |
| Stroke | 12(6.0) | 18(5.0) | 18(7.8) | 48(6.1) |
| Diarrheal | 9(4.5) | 31(8.7) | 6(2.6) | 46(5.8) |
| Unspecified infectious | 6(3.0) | 21(5.9) | 18(7.8) | 45(5.7) |
| Malaria | 8(4.0) | 27(7.6) | 6(2.6) | 41(5.2) |
| HIV/AIDS | 15(7.5) | 11(3.1) | 8(3.4) | 34(4.3) |
| Obstetric hemorrhage | 5(2.5) | 17(4.8) | 11(4.7) | 33(4.2) |
| Digestive neoplasms | 5(2.5) | 4(1.1) | 21(9.1) | 30(3.8) |
| Unspecified GI | 4(2.0) | 16(4.5) | 7(3.0) | 27(3.4) |
| Breast neoplasms | 10(5.0) | 9(2.5) | 2(0.9) | 21(2.7) |
| Unspecified neoplasms | 5(2.5) | 9(2.5) | 7(3.0) | 21(2.7) |
| Epilepsy | 8(4.0) | 4(1.1) | 5(2.2) | 17(2.2) |
| Acute abdomen | 7(3.5) | 6(1.7) | 3(1.3) | 16(2.0) |
| Renal failure | 5(2.5) | 6(1.7) | 4(1.7) | 15(1.9) |
| Liver cirrhosis | 3(1.5) | 6(1.7) | 5(2.2) | 14(1.8) |
| Unspecified maternal | 3(1.5) | 1(0.3) | 9(3.9) | 13(1.6) |
| Cause of death unknown | 25(12.6) | 48(13.4) | 34(14.7) | 107(1.6) |
